# Epidemiology of influenza B/Yamagata and B/Victoria lineages in South Africa, 2005-2014

**DOI:** 10.1371/journal.pone.0177655

**Published:** 2017-05-25

**Authors:** Mpho Seleka, Florette K. Treurnicht, Stefano Tempia, Orienka Hellferscee, Senzo Mtshali, Adam L. Cohen, Amelia Buys, Johanna M. McAnerney, Terry G. Besselaar, Marthi Pretorius, Anne von Gottberg, Sibongile Walaza, Cheryl Cohen, Shabir A. Madhi, Marietjie Venter

**Affiliations:** 1 Centre for Respiratory Diseases and Meningitis, National Institute for Communicable Diseases (NICD) of the National Health Laboratory Services (NHLS), Johannesburg, South Africa; 2 Influenza Division, Centers for Disease Control and Prevention, Atlanta, Georgia, United States of America; 3 Influenza Program, Centers for Disease Control and Prevention, Pretoria, South Africa; 4 School of Pathology, Faculty of Health Sciences, University of the Witwatersrand, Johanneburg, South Africa; 5 Sequencing Core Facility, National Institute for Communicable Diseases (NICD) of the National Health Laboratory Services (NHLS), Johannesburg, South Africa; 6 Global Influenza Program, World Health Organization (WHO), Geneva, Switzerland; 7 Medical Research Council, Respiratory and Meningeal Pathogens Research Unit, University of the Witwatersrand, Johannesburg, South Africa; 8 School of Public Health, Faculty of Health Sciences, University of the Witwatersrand, Johannesburg, South Africa; 9 Department of Science and Technology/National Research Foundation: Vaccine Preventable Diseases, University of the Witwatersrand, Johannesburg, South Africa; 10 Zoonoses Research Unit, Department of Medical Virology, University of Pretoria, Pretoria, South Africa; University of Hong Kong, HONG KONG

## Abstract

**Background:**

Studies describing the epidemiology of influenza B lineages in South Africa are lacking.

**Methods:**

We conducted a prospective study to describe the circulation of influenza B/Victoria and B/Yamagata lineages among patients of all ages enrolled in South Africa through three respiratory illness surveillance systems between 2005 and 2014: (i) the Viral Watch (VW) program enrolled outpatients with influenza-like illness (ILI) from private healthcare facilities during 2005–2014; (ii) the influenza-like illnesses program enrolled outpatients in public healthcare clinics (ILI/PHC) during 2012–2014; and (iii) the severe acute respiratory illnesses (SARI) program enrolled inpatients from public hospitals during 2009–2014. Influenza B viruses were detected by virus isolation during 2005 to 2009 and by real-time reverse transcription polymerase chain reaction from 2009–2014. Clinical and epidemiological characteristics of patients hospitalized with SARI and infected with different influenza B lineages were also compared using unconditional logistic regression.

**Results:**

Influenza viruses were detected in 22% (8,706/39,804) of specimens from patients with ILI or SARI during 2005–2014, of which 24% (2,087) were positive for influenza B. Influenza B viruses predominated in all three surveillance systems in 2010. B/Victoria predominated prior to 2011 (except 2008) whereas B/Yamagata predominated thereafter (except 2012). B lineages co-circulated in all seasons, except in 2013 and 2014 for SARI and ILI/PHC surveillance. Among influenza B-positive SARI cases, the detection of influenza B/Yamagata compared to influenza B/Victoria was significantly higher in individuals aged 45–64 years (adjusted odds ratio [aOR]: 4.2; 95% confidence interval [CI]: 1.1–16.5) and ≥65 years (aOR: 12.2; 95% CI: 2.3–64.4) compared to children aged 0–4 years, but was significantly lower in HIV-infected patients (aOR: 0.4; 95% CI: 0.2–0.9).

**Conclusion:**

B lineages co-circulated in most seasons except in 2013 and 2014. Hospitalized SARI cases display differential susceptibility for the two influenza B lineages, with B/Victoria being more prevalent among children and HIV-infected persons.

## Introduction

Influenza A and B viruses co-circulate globally during winter in the Northern and Southern hemispheres causing seasonal influenza epidemics, while epidemics are sporadic in the tropics [1–3]. Influenza B viruses were first identified in 1940 and, unlike influenza A, they cause disease primarily in humans [4, 5]. Studies have found that the symptoms and duration of illness due to infection with influenza A and influenza B are similar [6]. During the mid-1980’s, evolutionary changes in the haemagglutinin (HA) gene by antigenic drift separated influenza B viruses into two antigenically distinct lineages represented by B/Yamagata/16/88 and B/Victoria/2/87 [7]. Phylogenetic studies from different parts of the world demonstrated that influenza B viruses evolve through progressive antigenic drift at a slower rate compared with influenza A viruses [5, 7–9]. Influenza B disease burden has been reported to be higher in children (0–17 years old) and young adults (25–44 years old) [10–15]. Studies conducted in South Africa reported that the incidence of influenza B-associated severe acute respiratory illness (SARI) hospitalization was higher among HIV-infected persons compared to HIV-uninfected persons [16, 17].

The Southern hemisphere formulation of inactivated trivalent influenza vaccine (TIV), consisting of one influenza B lineage and two influenza A strains, is available annually in South Africa [18]. Vaccine coverage was <5% for the 2005–2014 period, with the highest coverage in the high risk groups with underlying medical conditions [19–21]. The recently published national influenza policy for South Africa also highlights the following groups at high risk for severe influenza to be prioritized for influenza vaccination: the very young (healthy and unhealthy children), the elderly, pregnant women and persons with underlying medical conditions including persons with HIV and TB [22]. In South Africa it is unclear if any of the described categories of persons at high risk for severe influenza virus infection display differential susceptibility for infection with the two influenza B lineages.

We aim to describe the circulating patterns of influenza B/Victoria and B/Yamagata strains in South Africa, a country with a high HIV a burden using influenza sentinel surveillance data obtained from outpatients with influenza-like illness (ILI) (2005–2014) and inpatients with SARI (2009–2014). In addition we aim to describe the clinical and epidemiological factors associated with influenza B/Victoria and B/Yamagata-positive patients hospitalized with SARI in South Africa during 2009–2014.

## Materials and methods

### Surveillance programs

We conducted inpatient and outpatient prospective surveillance by collecting demographic and clinical data, and respiratory samples using three respiratory illness surveillance programs [23, 24]. The Viral Watch (VW) program is a sentinel surveillance program initiated in 1984 for outpatients with ILI seen by private healthcare providers [25]. Participation in the program is voluntary and as such patients’ enrolment is often based on the need for laboratory diagnosis as part of clinical case management. Therefore patient enrolment is not systematic, with majority of the specimens submitted during the winter period (May-September) when influenza virus is expected to circulate. By 2008, all 9 provinces of the country were taking part in the VW program [25]. Criteria for enrolment included patients presenting at sentinel healthcare providers with a measured temperature of ≥38°C or a history of fever, and cough, with onset within the past 7 days before 2013 and 10 days from 2013 onwards (duration of symptoms was changed to 10 days in 2013 in line with World Health Organization (WHO) Global Influenza Surveillance Guidelines, published January 2014) [25, 26]. During 2005–2008 limited demographic and clinical information was collected on enrolled VW patients. From 2010 information on underlying medical conditions was also recorded.

The SARI surveillance program is a year-round, active sentinel hospital-based surveillance system which started in 2009 in three provinces and from 2010 it covered 4/9 provinces of South Africa [16]. Age-specific case definitions were used to systematically recruit patients who presented with symptom duration ≤7 days (changed to ≤10 days in 2013): (i) infants aged 2 days to <3 months with a diagnosis of suspected sepsis or physician-diagnosed lower respiratory tract infection (LRTI) irrespective of signs and symptoms; (ii) children 3 months to <5 years hospitalized with physician-diagnosed LRTI including bronchitis, bronchiolitis, pneumonia and pleural effusion; (iii) individuals ≥5 years old with sudden onset of fever (≥38°C or history of fever) and cough or sore throat and shortness of breath or difficult breathing with or without clinical or radiographic findings of pneumonia [16, 24, 26, 27]. Detailed demographic and clinical data were collected for each patient enrolled by administering a standardized questionnaire and performing hospital record reviews [27]. HIV status of patients were determined through a combination of sources for HIV results including: (i) patient clinical records, (ii) for consenting patients, anonymized linked dried blood spot testing at the National Institute for Communicable Diseases (NICD) or (iii) pre-test counselling and bedside rapid testing for HIV. The NICD HIV test results were the preferred results when available.

An active sentinel surveillance program for ILI in public health clinics (ILI/PHC) was initiated in May 2012 in 2/4 provinces where SARI surveillance was also conducted. It is a year-round systematic surveillance program, unlike VW. An ILI case was defined as an outpatient of any age presenting with either temperature ≥38°C or history of fever and cough of duration of ≤7 days (changed to ≤10 days in 2013) [26]. Similar to SARI, detailed demographic and clinical data as well as HIV status were recorded for each patient enrolled.

### Study specimens

Throat and/or nasal swabs were collected from patients of all ages under the VW surveillance program [25]. For the SARI and ILI/PHC surveillance programs, nasopharyngeal aspirates (NPA) were mainly collected from children <5 years of age, whereas combined nasopharyngeal (NP) and oropharyngeal (OP) swabs were collected from patients ≥5 years of age [28, 29]. Respiratory specimens including nasopharyngeal aspirates were collected in viral transport medium (Highveld Biological, Johannesburg, South Africa) or universal transport medium (Copan, Murrieta, California, USA) and transported on ice to reach the laboratory within 72 hours of collection.

Respiratory specimens from patients enrolled from all surveillance programs for the period 2005 to 2014 were used to determine the seasonal proportions of influenza B virus infections and to determine the seasonal circulation patterns for B/Victoria and B/Yamagata lineages. Only participants enrolled from the SARI program were used to determine factors associated with influenza B lineages infection.

### Influenza virus isolation and serotyping

Virus isolation was the primary diagnostic assay used for influenza virus detection during the 2005 to mid-2009 pre-pandemic period for the VW program. Respiratory specimens were treated with antibiotics and 200 μμl was inoculated onto Madin-Darby Canine Kidney (MDCK) cell monolayers either in flasks or on shell vials (ThermoFisher Scientific, Waltham, Massachusetts, USA). Cultures were observed daily and once cytopathic effect (CPE) became apparent, cytospin preparations using polyclonal and strain specific fluorescein-conjugated monoclonal antibody staining (Merck Millipore Corporation, Darmstadt, Germany) for confirmation of influenza A or B infection by immunofluorescence microscopy was performed [30].

Influenza A and B virus positive isolates from 2005–2009 were serotyped with strain-specific hyper-immune sera raised in rabbits or sheep using haemagglutination inhibition (HAI) assay to determine identity to the seasonal vaccine strains or reference viruses. Influenza serotyping kits were supplied by the World Health Organization Collaborating Centre (WHO-CC) for Influenza Surveillance and Research, Melbourne, Australia [31–33].

### Real-time reverse transcription polymerase chain reaction (RT-PCR) assays for influenza virus detection and lineage-subtyping

#### RT-PCR assay for influenza virus detection

Respiratory specimens submitted through the VW, SARI and ILI/PHC programs from 2009 onwards were tested for influenza using the CDC Influenza A/B Virus Real-Time RT-PCR Diagnostic Typing Kit [34, 35]. Briefly, the assay reaction was performed using Invitrogen Superscript^®^ III Platinum^®^ One-Step RT-PCR (ThermoFisher Scientific, Waltham, Massachusetts, USA) or Ambion AgPath-ID^™^ One-Step RT-PCR Kit (ThermoFisher Scientific, Waltham, Massachusetts, USA) buffer and enzyme systems. Applied Biosystems^™^ 7500 Fast (ThermoFisher Scientific, Waltham, Massachusetts, USA) or the LightCycler^®^ 480 (Roche Diagnostics, Mannhein, Germany) were used as real-time detection systems and a total of 40 cycles were run.

#### Real-time RT-PCR assays for differentiation of influenza B lineages

Influenza B lineage typing was done retrospectively on respiratory specimens submitted during mid-2009 to 2014 for all surveillance programs and tested positive for influenza B virus. Influenza B positive specimens from 2009–2011 were distinguished into influenza B Yamagata and Victoria lineages by real-time RT-PCR using the TaqMan probes as described [36, 37]. From 2012, influenza B positive specimens were typed using the CDC real-time one-step RT-PCR lineage typing kits obtained through the International Reagent Resource Program (IRR, https://www.internationalreagentresource.org) and the protocol as previously described [38]. The assay protocol was similar to the CDC Influenza A/B typing and influenza A subtyping protocols validated to use with either Invitrogen SuperScript^™^ III Platinum^®^ One-step Quantitative RT-PCR System (ThermoFisher Scientific, Waltham, Massachusetts, USA) or Ambion AgPath-ID^™^ One-Step RT-PCR Kit (ThermoFisher Scientific, Waltham, Massachusetts, USA) enzyme systems.

### Statistical analysis

#### Proportion of influenza B positives from all surveillance programs

The proportion of influenza B positives among influenza virus positive specimens was determined in all years for all surveillance programs separately. The proportion of influenza B/Victoria and B/Yamagata circulating in each year was also determined among specimens that could be typed. An influenza B positive specimen was considered lineage-typed if an influenza B lineage (B/Yamagata or B/Victoria) could be assigned. The proportion of circulating influenza B viruses mismatched to the vaccine strain was calculated based on the total number of successfully lineage-typed specimens.

#### Factors associated with influenza B lineage infection among patients with SARI

We used unconditional logistic regression to assess factors associated with influenza B lineage-specific infections among influenza B positive patients hospitalized with SARI. For the multivariable model we assessed all variables with p value <0.2 on univariate analysis, and dropped non-significant factors (p>0.05) with manual backward elimination [27, 39]. Only variables significant at p<0.05 were retained in the multivariable model. Variables with p<0.05 were considered significant at univariate or multivariable analysis. Pairwise interactions were assessed by inclusion of product terms for all variables remaining in the final multivariable additive model.

#### Proportion of lineage-typed influenza B specimens by Ct-value among influenza B positive patients with SARI

Because we were unable to lineage type a high proportion of the influenza B positive specimens (range from 9% to 79% per season), we implemented a sensitivity analysis for the lineage typing. In this analysis we evaluated the proportion of influenza B lineage-typed specimens by influenza B Ct-value. We used logistic regression to compare the proportion of influenza B-positive lineage-typed specimens with Ct-values ≤30 to the proportion of lineage-typed specimens with individual Ct-values from 31–40. P-values <0.05 were considered to be statistically significant.

All statistical analyses were performed using STATA version 14.1 (Stata Corporation, College Station, Texas USA).

### Ethical approval

The SARI protocol was approved by the University of the Witwatersrand Human Research Ethics Committee (HREC) and the University of KwaZulu-Natal Human Biomedical Research Ethics Committee (BREC) protocol numbers M081042 and BF157/08, respectively. The ILI surveillance protocol in public health clinics was approved by HREC and BREC protocol numbers M120133 and BF080/12, respectively.

VW surveillance specimens were taken from patients as part of routine diagnostic investigations for which written informed consent was not required. Essential communicable disease surveillance activities of the NICD that are of national public health importance are covered by ethical clearance certificate number M110499 issued by University of the Witwatersrand Human Research Ethics Committee. This surveillance was deemed non-research by the U.S. Centres for Disease Control and Prevention.

## Results

### Study population and proportion of influenza B positives from 2005 to 2014

During the study period a total of 39,804 patients were enrolled and tested for influenza, of which 16,146 (40.6%) were from the VW program (2005–2014), 18, 303 (46%) were from the SARI program (2009–2014) and 5,355 (13.4%) were from the ILI/PHC program (2012–2014) (Table 1). Among patients with available information, patients of black race accounted for 97.9% (17,815/18,201) of SARI cases and 98.9% (5,218/5,277) of ILI cases enrolled in ILI/PHC, whereas race was not recorded in the VW program. Females accounted for 52.9% (8,487/16,029), 50.4% (9,189/18,231) and 66.9% (3,532/5,282) of patients in the VW, SARI and ILI/PHC study populations, respectively. Children <5 years of age accounted for 10% (1,619/16,110), 56.6% (10,315/18,229) and 20.3% (1,087/5,352) of the study participants in the VW, SARI and ILI/PHC, respectively. The prevalence of underlying medical conditions was 11.5% (2,089/18,211) and 19.3% (1,021/5,276) in SARI and ILI/PHC study population, respectively. Underlying medical conditions was not well defined in VW. The HIV prevalence was 1.9% (150/7,905), 40.9% (5,798/14,189) and 36.1% (1,774/4,912) in the VW, SARI and ILI/PHC study population, respectively. Influenza vaccine coverage was 4.9% (696/14,163), 0.2% (34/17,827) and 1.7% (92/5,262) in the VW, SARI and ILI/PHC surveillance programs, respectively.

**Table 1:** Detection rates for influenza viruses in the three surveillance program, Viral Watch (VW), Illnesses-like influenza surveillance in public health clinics (ILI/PHC) and severe acute respiratory illnesses (SARI) in South Africa, 2005–2014.

Surveillance program	Year	^a^Influenza A/B positives n/N (%)	influenza B positives N (%)	influenza B untyped N (%)	B/Victoria ^b^N (%)	B/Yamagata ^b^N (%)	^c^Seasonal Trivalent Influenza Vaccine lineage (name)
**VW (Virus isolation and serotyping)**	2005	564/1358 (42)	106 (19)	3(3)	88 (85)	15 (15)	B/Yamagata (B/Shanghai/361/2002)*
	2006	491/1219 (40)	39 (8)	2 (5)	31 (84)	6 (16)	B/Victoria (B/Malaysia/2506/2004)
	2007	468/1389 (34)	139 (30)	6(4)	78 (57)	58 (43)	B/Victoria (B/Malaysia/2506/2004)
	2008	300/1276 (24)	33 (11)	2 (6)	5 (16)	26 (84)	B/Yamagata (B/Florida/4/2006)
	**Total**	**1823/5242 (35)**	**317 (17)**	**13 (4)**	**202 (66)**	**105 (34)**	
**VW (Virus isolation/ serotyping and real-time PCR)**	2009^a^	932/2074 (45)	46 (5)	21 (46)	17 (67)	8 (33)	B/Yamagata (B/Florida/4/2006)*
**VW (Real-time PCR)**	2010	827/1926 (43)	445 (54)	320(72)	116 (93)	9 (7)	B/Victoria (B/Brisbane/60/2008)
	2011	986/2581 (38)	118 (12)	93 (79)	6 (24)	19 (76)	B/Victoria (B/Brisbane/60/2008)*
	2012	576/1468 (39)	236 (41)	52 (22)	146 (79)	38 (21)	B/Victoria (B/Brisbane/60/2008)
	2013	842/1865 (45)	114 (14)	34 (30)	4 (5)	76 (95)	B/Yamagata (B/Wisconsin/1/2010)
	2014	502/990 (51)	76 (15)	7 (9)	3 (4)	66 (96)	B/Yamagata (B/Massachusetts/2/2012)
	**Total**	**3733/8830 (42)**	**989(26)**	**506 (51)**	**275 (57)**	**208 (43)**	
**VW**	**Grand total**	**6488/16 146 (40)**	**1352 (21)**	**540 (40)**	**494 (61)**	**321 (39)**	
**ILI/PHC (Real-time PCR)**	2012	215/1028 (21)	142 (66)	6 (4)	109 (80)	27 (20)	B/Victoria (B/Brisbane/60/2008)
	2013	243/1975 (12)	55 (23)	12 (22)	0 (0)	43 (100)	B/Yamagata (B/Wisconsin/1/2010)
	2014	311/2352 (13)	54 (17)	12 (22)	0 (0)	42 (100)	B/Yamagata (B/Massachusetts/2/2012)
**ILI/PHC**	**Grand total**	**769/5355 (14)**	**251 (33)**	**30 (12)**	**109 (49)**	**112 (51)**	
**SARI (Real-time PCR)**	2009	420/3661 (11)	28 (7)	9 (32)	15 (79)	4 (21)	B/Yamagata (B/Florida/4/2006)*
**SARI (Real-time PCR)**	2010	279/3940 (7)	179 (64)	105 (59)	70 (95)	4 (5)	B/Victoria (B/Brisbane/60/2008)
	2011	366/4203 (9)	128 (35)	53 (41)	13 (17)	62 (83)	B/Victoria (B/Brisbane/60/2008)*
	2012	231/3889 (6)	122 (53)	23 (19)	83 (84)	16 (16)	B/Victoria (B/Brisbane/60/2008)
	2013	113/1802 (6)	20 (18)	4 (20)	0 (0)	16 (100)	B/Yamagata (B/Wisconsin/1/2010)
	2014	40/808 (5)	7 (18)	4 (57)	0 (0)	3 (100)	B/Yamagata (B/Massachusetts/2/2012)
	**Total**	**1029/14 642 (7)**	**456 (44)**	**189 (41)**	**166 (62)**	**101 (38)**	
**SARI**	**Grand total**	**1449/18 303 (8)**	**484 (33)**	**198 (41)**	**181 (63)**	**105 (37)**	

^a^Number of influenza positive specimens out of a total screened for influenza A and B virus per year.

^b^N stands for the total number of influenza B positives that could be characterized into lineages per year.

^c^Lineage (and name) of influenza B virus included in the Southern Hemisphere seasonal vaccines which were retrieved from http://www.who.int/influenza/vaccines/virus/recommendations/en/.

*Mismatched years

**Fig 1:**
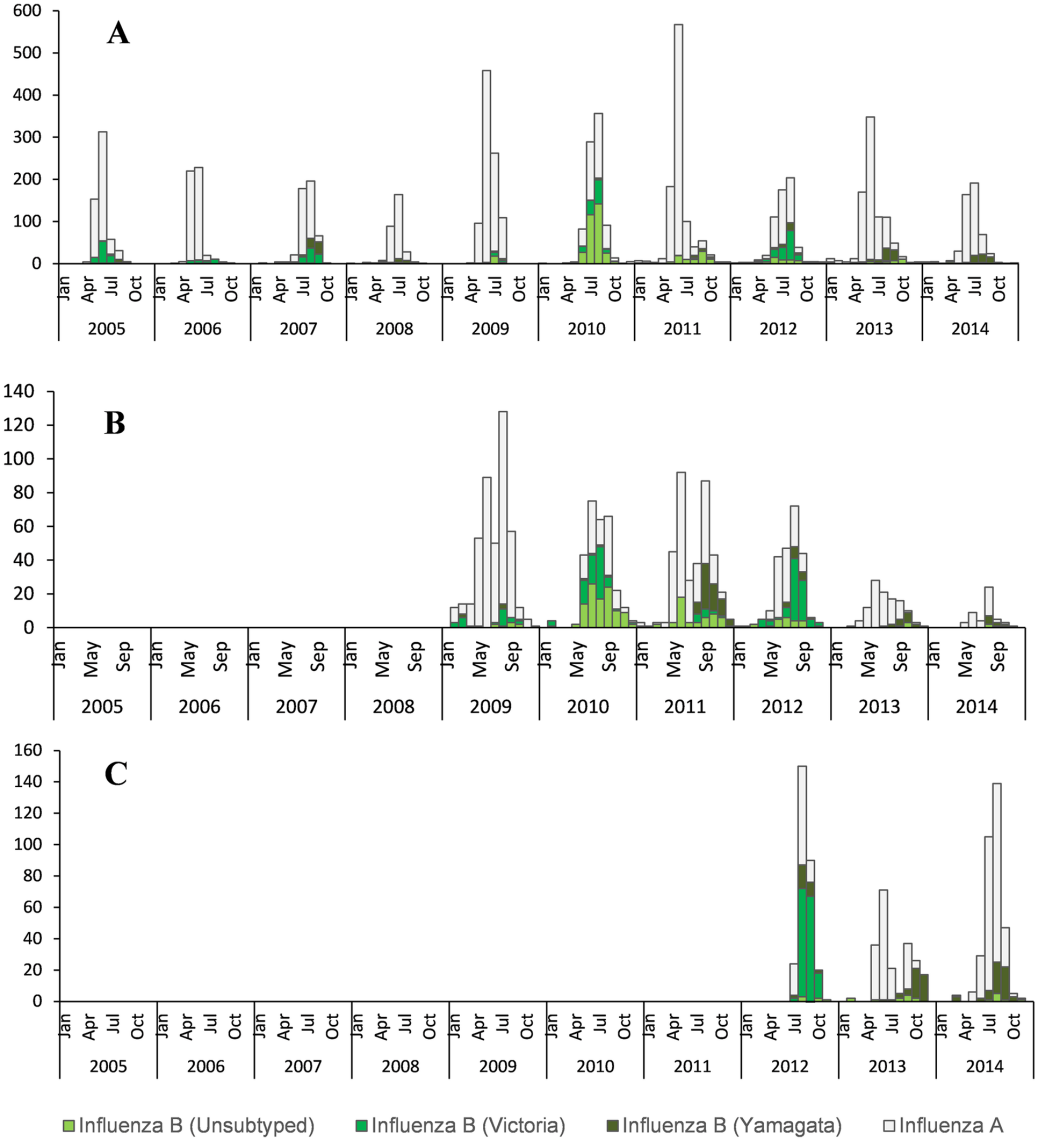
Monthly number of influenza-positive specimens in South Africa. (**A**) Viral Watch program (2005–2014), (**B**) Severe acute respiratory illness surveillance program (2009–2014), and (**C**) Influenza-like illness surveillance program in public health clinics (2012–2014).

The overall influenza A/B virus detection rate over the 10-year period was 21.9% (8,706/39,804) in specimens sampled from patients enrolled in all three surveillance programs. The detection rate was higher in the VW program (40.2%; 6,489/16,146) than in the SARI (7.9%; 1,449/18,303) and ILI/PHC (14.4%; 769/5,355) programs (Table 1). Over the study period influenza A viruses were detected at higher frequencies than influenza B in all three surveillance programs, except in 2010. In 2012 influenza B viruses were detected at higher frequencies than influenza A viruses in SARI and ILI/PHC (Table 1). In general, influenza A virus detection peaked in June-July for the VW program, compared to June-August for the SARI and ILI programs (Fig 1).

Influenza B viruses accounted for 24% (2,087/8,706) of influenza positive specimens. The frequency of influenza B detections over the study period was 20.8% (1,352/6,488) for VW (2005–2014), 33.4% (484/1,449) for SARI (2009–2014) and 32.6% (251/769) for ILI/PHC (2012–2014). Generally, influenza B viruses tended to circulate at lower frequencies towards middle or end of the influenza season with peaks in August-September (Fig 1).

### Annual circulation of influenza B/Victoria and B/Yamagata lineages

Overall, of 2,087 influenza B positive samples, 1,322 (63.3%) could be lineage typed. Of these, 784 (59.3%) were B/Victoria and 538 (40.6%) were B/Yamagata lineage. The two lineages co-circulated in the VW program in all seasons. In SARI and ILI/PHC surveillance it also co-circulated in almost all seasons, except 2013 and 2014 when only B/Yamagata viruses were detected (Table 1). In the systematic ILI/PHC and SARI surveillance programs B/Victoria lineage viruses were detected at overall frequencies of 49.3% (109/221) and 63.3% (181/286), respectively. Irrespective of surveillance program, B/Victoria was the most frequently detected lineage in 6 of the 10 study years. Influenza B/Victoria predominated from 2005 to 2012 with the exception of 2008 and 2011 (Table 1 and Fig 1).

### Factors associated with influenza B lineages among patients with SARI

During 2009–2014 influenza B viruses were detected in a total of 484 SARI cases, of which >90% had complete data for the factors evaluated in this analysis (Table 2). After adjusting for year, on multivariable logistic regression analysis the detection of influenza B/Yamagata compared to influenza B/Victoria was significantly higher in older individuals aged 45–64 years (adjusted odds ratio [aOR]: 4.2; 95% confidence interval [CI]: 1.1–16.5) and ≥65 years (aOR: 12.2; 95% CI: 2.3–64.4) compared to 0–4 years, but was significantly lower in HIV-infected patients (aOR: 0.4; 95% CI: 0.2–0.9) (Table 2). No association was found with other underlying medical conditions, duration of symptoms, hospital stay or disease progression.

**Table 2:** Demographic and clinical characteristics associated with influenza B lineages, determined by multivariable logistic regression analysis, among influenza B-positive patients hospitalized with severe acute respiratory illness, South Africa, 2009–2014.

Characteristics of Patients	Influenza B n/N^b^ (%)	B/Victoria n/N^c^ (%)	B/Yamagata n/N^c^ (%)	Untyped B n/N^d^ (%)	Odd Ratio (95% CI)	P-value	Adjusted Odd ratio (95% CI)	P-value
**Year**^a^								
2009	28/484 (5.8)	15/181 (8.3)	4/105 (3.8)	9/198 (4.5)	Reference	-	Reference	-
2010	179/484 (37.0)	70/181 (38.7)	4/105 (3.8)	105/198 (53)	0.2 (<0.1–0.9)	**0.036**	0.2 (<0.1–1.1)	0.057
2011	128/484 (26.4)	13/181 (7.2)	62/105 (59.0)	53/198 (26.8)	15.9 (4.8–53.1)	**<0.001**	11.8 (3.1–45.7)	**<0.001**
2012	122/484 (25.2)	83/181 (45.9)	16/105 (15.2)	23/198 (11.6)	0.7 (0.2–2.2)	0.521	0.5 (0.1–2.0)	0.327
2013	20/484 (4.1)	0/181 (0.0)	16/105 (15.2)	4/198 (2)	113.7 (5.6–234.2)	**0.002**	73.7 (3.3–186.4)	**0.007**
2014	7/484 (1.4)	0/181 (0.0)	3/105 (2.9)	4/198 (2)	24.1 (1.1–53.7)	**0.047**	9.7 (0.3–17.6)	0.189
**Age (in years)**								
0–4	199/482 (41.3)	87/181 (48.1)	39/104 (37.5)	73/197 (37.1)	Reference	-	Reference	-
5–24	47/482 (9.8)	19/181 (10.5)	11/104 (10.6)	17/197 (8.6)	1.3 (0.6–3.0)	0.547	1.6 (0.4–6.5)	0.526
25–44	146/482 (30.3)	54/181 (29.8)	32/104 (30.8)	60/197 (30.5)	1.3 (0.7–2.4)	0.344	2.0 (0.6–6.2)	0.233
45–64	68/482 (14.1)	18/181 (9.9)	14/104 (13.5)	36/197 (18.3)	1.7 (0.8–3.8)	0.174	4.2 (1.1–16.5)	**0.038**
≥65	22/482 (4.6)	3/181 (1.7)	8/104 (7.7)	11/197 (5.6)	5.9 (1.5–23.6)	**0.011**	12.2 (2.3–64.4)	**0.003**
**Sex (Female)**	278/483 (57.6)	106/181 (58.6)	63/104 (60.6)	109/198 (55.1)	1.1 (0.7–1.8)	0.739		
**Underlying medical conditions**								
Any	52/483 (10.8)	24/181 (13.3)	14/104 (13.5)	14/198 (7.1)	1.0 (0.5–2.1)	0.962		
Asthma	16/483 (3.3)	7/181 (3.9)	5/104 (4.8)	4/198 (2)	1.3 (0.4–4.1)	0.704		
Chronic lung disease	1/483 (0.2)	1/181 (0.6)	0/104 (0.0)	0	1.7 (0.0–67.8)	1.000		
Chronic heart disease	2/483 (0.4)	1/181 (0.6)	0/104 (0.0)	1/198 (0.5)	1.7 (0.0–67.8)	1.000		
Diabetes	11/483 (2.3)	2/181 (1.1)	5/104 (4.8)	4/198 (2)	4.5 (0.9–23.7)	0.075		
Obesity	3/483 (0.6)	2/181 (1.1)	1/104 (1.0)	0	0.9 (0.1–9.7)	0.909		
HIV infection	194/405 (47.9)	65/145 (44.8)	34/90 (37.8)	95/170 (55.9)	0.7 (0.4–1.3)	0.288	0.4 (0.2–0.9)	**0.047**
**Duration of symptoms ≥3 (in days)**	273/480 (56.9)	100/180 (55.6)	56/104 (53.8)	117/196 (59.7)	0.9 (0.6–1.5)	0.780		
**Duration of hospitalization (in days)**								
<3	145/470 (30.9)	59/177 (33.3)	34/98 (34.7)	52/195 (26.7)	Reference			
3–7	221/470 (47.0)	84/177 (47.5)	48/98 (49.0)	89/195 (45.6)	1.0 (0.6–1.7)	0.976		
≥8	104/470 (22.1)	34/177 (19.2)	16/98 (16.3)	54/195 (27.7)	0.8 (0.4–1.7)	0.586		
**Progression of illness and in-hospital outcome**								
Died	19/475 (4.0)	5/179 (2.8)	5/99 (5.1)	9/197 (4.6)	1.9 (0.5–6.6)	0.340		
ICU admission	4/475 (0.8)	3/178 (1.7)	0/100 (0.0)	1/197 (0.5)	0.5 (0.0–4.3)	0.522		
Oxygen therapy	168/475 (35.4)	59/178 (33.1)	31/100 (31.0)	78/197 (39.6)	0.9 (0.5–1.5)	0.714		

^a^ Estimate using penalized logistic regression.

^b^Total number of influenza B viruses detected in the SARI cases of which n had the respective data available.

^c^Total number of influenza B/Victoria or B/Yamagata viruses detected in SARI cases of which n had the respective data.

^d^Total number of untyped influenza B positive cases of which n had the respective data.

### Proportion of influenza B lineage-typed specimens by influenza B Ct-value among influenza B positive patients with SARI

Of the 18, 303 SARI cases enrolled and tested during 2009–2014, 1,449 (7.9%) tested positive for influenza virus infection, of which 484 (33.4%) were influenza B positive (Table 1). Overall, 59.1% (286/484) of influenza B positive specimens could be distinguished as lineage B/Victoria or B/Yamagata. Compared to specimens with influenza B Ct-value ≤30, we observed a significant decline in the proportion of lineage-typed samples that had an individual influenza B Ct-value ≥36 (Table 3). The proportion of lineage-typed specimens progressively declined from 89.0% (154/173) among those with Ct-values ≤30 to 12.7% (8/63) among those with Ct-value of 40 (p<0.001). A statistically significant difference was observed in the proportion of influenza B-positive specimens with Ct-values ≥36 by year: 12% (3/25) in 2009, 54% (81/149) in 2010, 43% (55/127) in 2011, 25% (31/122) in 2012, 15% (3/20) in 2013 and 60% (3/5) in 2014 (p<0.001).

**Table 3:** Proportion of typed samples by influenza B Ct-value among patients with SARI.

SARI Influenza B Ct-value	Typed n/N (%)	OR (95% CI)	p-value
**30**	154/173 (89.0)	Reference	
**31**	22/29 (75.9)	0.4 (0.1–1.0)	0.057
**32**	12/14 (85.7)	0.7 (0.2–3.6)	0.707
**33**	11/12 (91.7)	1.4 (0.2–11.1)	0.776
**34**	17/20 (85.0)	0.7 (0.2–2.6)	0.594
**35**	19/24 (79.2)	0.5 (0.2–1.4)	0.175
**36**	17/29 (58.6)	0.2 (0.1–0.4)	0.000
**37**	8/15 (53.3)	0.1 (0.0–0.4)	0.001
**38**	7/23 (30.4)	0.1 (0.0–0.1)	0.000
**39**	5/46 (10.9)	0.0 (0.0–0.0)	0.000
**40**	8/63 (12.7)	0.0 (0.0–0.0)	0.000

## Discussion

We reported the circulation patterns of influenza B virus and its lineages a 10-year period in South Africa. Overall, influenza B viruses accounted for about 24% of influenza positive cases, with B/Victoria and B/Yamagata viruses detected at frequencies of 59.3% and 40.7%, respectively, among influenza B-positive samples that could be lineage-typed. This is similar to global findings (average of ≈25% influenza B, 64% B/Victoria and 36%) for the 2000–2013 period [40]. Influenza B viruses rarely dominate over influenza A viruses and it is reportedly observed only once in every 10 seasons [15]. Other Southern Hemisphere countries reported average influenza B virus detection below 20% for influenza-positive cases across multiple seasons: Brazil (19%: 2006–2014), Australia (17.1%: 2001–2014) and New Zealand (16.8%: 2000–2012); whereas Southern China (2006–2012) reported a higher frequency of 29.4% [40–42]. Northern Hemisphere countries such as United States (16%: 2000–2012), Ukraine (22.9%: 2000–2012), England (25%: 2003–2013) and Finland (26%: 1999–2012) reported influenza B frequencies similar to global studies, but a higher average circulation frequency of 31% for Northern China (2005–2012) [13, 40].

Of the 10 influenza seasons covered in our study period the influenza B viruses circulated at higher frequencies than influenza A viruses only in 2010 across all three surveillance programs. Additionally, in 2012 influenza B circulated at higher frequencies in SARI and ILI/PHC surveillance, which is consistent with published literature from other countries [15, 43]. Influenza B/Victoria was the predominant lineage circulating in 2010 and 2012 at frequencies ranging between 79–96%, matching the vaccine strain type (see Table 1). B/Victoria viruses were dominant over the B/Yamagata viruses in 6 out of 10 seasons, similar to Taiwan (Northern Hemisphere country) who reported dominant circulation of B/Yamagata in 2004/2005 and 2013/2014 seasons [44]. In contrast to South Africa, in Brazil B/Yamagata lineage strains dominated in 2005, 2009, 2010 and B/Victoria lineages in 2011–2013 [12].

Influenza Vaccine coverage in our study population, especially among hospitalized patients, was low. The influenza B vaccine strain lineage included in the annual Southern Hemisphere vaccine is reported in Table 1. The Southern Hemisphere influenza B vaccine strain was mismatched to the predominant circulating influenza B viruses in South Africa in only 3/10 seasons (2005, 2009 and 2011). B/Victoria was the predominant circulating lineage in 2/3 seasons mismatched to vaccine at frequencies above 70% (2005: 85% and 2009: 73%) and in 2011 B/Yamagata lineage circulated at a frequency of 81%. In Brazil, the influenza B vaccine strain was mismatched to the predominant circulating lineage (for the period 2006–2014) in the 2013 season at a frequency of 91.4% [41]. Australia reported 2 seasons (2007 and 2009) mismatched (>60% of circulating B lineage) to the vaccine strain over the same study period [42]. In contrast, Northern Hemisphere countries reported more seasons where the circulating influenza B viruses were mismatched to the seasonal vaccine strain over the same period, namely; Taiwan, 5 seasons during 2005–2014, [44] and Finland, 4 during 2005–2012 [13].

We observed no co-circulation of the two lineages in 2013 and 2014 for the systematic ILI/PHC and SARI surveillance programs; in which only B/Yamagata viruses were detected. Co-circulation of the 2 influenza B lineages in VW could be linked to the program’s inclusion of a healthcare clinic at a port of entry to South Africa (domestic and international travel). Over the study period, during low influenza B seasons, these viruses were detected mainly towards the end of the influenza season.

In our study, B/Victoria detection was significantly associated with younger age compared to B/Yamagata among hospitalized SARI cases. This was also observed in studies conducted in Australia, New Zealand, Slovenia, Finland, Southern China and Brazil which showed that B/Victoria strains are predominantly detected among younger age groups [10–13, 42, 45]. In addition, B/Victoria detection was more common among HIV-infected individuals. The reason for the observed associations is unclear. Vijaykrishan et al [10] demonstrated that B/Victoria viruses, like the A(H3N2) viruses, undergo genetic drift and frequent selective bottlenecks whereby the circulating viruses are replaced [10]. This may suggest an increased fitness of the B/Victoria viruses and potentially higher transmission rates which enable them to readily infect very young children and immune-compromised persons. Alternatively, this might explain that this is due to older, immunocompetent adults likely having greater lifetime exposure to a variety of influenza viruses which may provide broader immunity to newer strains.

In our study, influenza B positive specimens with Ct values ≥36 were significantly less likely to be distinguished by their relevant influenza B lineages indicating that the lineage typing assay is less sensitive than the influenza B virus detection assay. These observations validated our updated laboratory Ct cut-off value of ≤37 for the diagnosis of influenza A and B by real-time RT-PCR typing assay which were implemented in October 2014. This cut-off will ensure repeatability of diagnostic results especially when specimens are shared between laboratories and when subtyping or genotyping of viruses are required.

Our study has limitations that warrant discussion. Despite attempts at standardization, specimen collection in the VW program is not always systematic due to the voluntary nature of the participation. This may underestimate the proportional contribution of influenza B especially if influenza B circulated more towards the end of the influenza season when the number of specimens collected under the VW program is low. Nonetheless, for years in which VW, SARI and ILI/PHC data were available, the dominant circulating influenza B lineage was concordant across all three programs. VW and ILI/PHC data were not used to determine factors associated with influenza B lineages because the former is not a systematic surveillance system and most specimens were submitted during the influenza season, and the latter did not have enough data since it started in 2012. We were also not powered to assess differences between geographical areas. We did not have data on the proportion of foreigners and resident subjects enrolled in any of our surveillance programs and data collected at sentinel sites may not be representative of the country. Influenza virus detection was mainly dependent on virus isolation until 2009. Therefore, the overall detection rates between 2005 and 2009 might have been underestimated as virus isolation methods are less sensitive than real-time RT-PCR.

In conclusion, this study suggests that there is differential age susceptibility for the two influenza B lineages, with B/Victoria being more prevalent among children compared to B/Yamagata. Similarly to young children, HIV-infected persons were the only high risk group prioritized for influenza vaccination in South Africa that showed increased susceptibility to infection with influenza B/Victoria lineage viruses.
